# Adaptation of a Digital Health Innovation to Prevent Relapse and Support Recovery in Youth Receiving Services for First-Episode Psychosis: Results From the Horyzons-Canada Phase 1 Study

**DOI:** 10.2196/19887

**Published:** 2020-10-29

**Authors:** Shalini Lal, John Gleeson, Lysanne Rivard, Simon D'Alfonso, Ridha Joober, Ashok Malla, Mario Alvarez-Jimenez

**Affiliations:** 1 School of Rehabilitation Faculty of Medicine University of Montréal Montréal, QC Canada; 2 Youth Mental Health and Technology Lab Health Innovation and Evaluation Hub University of Montréal Hospital Research Centre Montréal, QC Canada; 3 Prevention and Early Intervention Program for Psychosis (PEPP-Montreal) and ACCESS Open Minds Douglas Mental Health University Institute Montréal, QC Canada; 4 Healthy Brain and Mind Research Centre and School of Behavioural and Health Sciences Australian Catholic University Fitzroy Australia; 5 School of Computing and Information Systems The University of Melbourne Parkville Australia; 6 Department of Psychiatry McGill University Montréal, QC Canada; 7 Centre for Youth Mental Health The University of Melbourne Parkville Australia; 8 Orygen Parkville Australia

**Keywords:** psychotic disorders, mental health, telemedicine, young adult, mental health services, cultural adaptation, mobile phone, e-mental health, virtual care, schizophrenia, e-health

## Abstract

**Background:**

Developing a digital health innovation can require a substantial amount of financial and human resource investment before it can be scaled for implementation across geographical, cultural, and health care contexts. As such, there is an increased interest in leveraging eHealth innovations developed and tested in one country or jurisdiction and using these innovations in local settings. However, limited knowledge exists on the processes needed to appropriately adapt digital health innovations to optimize their transferability across geographical, cultural, and contextual settings.

**Objective:**

We report on the results of an adaptation study of Horyzons, a digital health innovation originally developed and tested in Australia. Horyzons is designed to prevent relapses and support recovery in young people receiving services for first-episode psychosis (FEP). The aim of this study is to assess the initial acceptability of Horyzons and adapt it in preparation for pilot testing in Canada.

**Methods:**

This research took place in 2 specialized early intervention clinics for FEP, located in 1 urban and 1 urban-rural setting, in 2 Canadian provinces. A total of 26 participants were recruited: 15 clinicians (age range 26-56 years) and 11 patients (age range 19-37 years). Following the digital health adaptation framework developed by our team, we used a mixed methods approach, combining descriptive quantitative and qualitative methods across 3 stages of data collection (focus groups, interviews, and consultations), analysis, and adaptations.

**Results:**

Overall, patients and clinicians appreciated the strengths-based approach and social media features of Horyzons. However, participants expressed concerns related to implementation, especially in relation to capacity (eg, site moderation, crisis management, internet speed in rural locations). They also provided suggestions for adapting content and features, for example, in relation to community resources, volume of text, universal accessibility (eg, for individuals with limitations in vision), and optimization of platform accessibility through mobile devices. Additional aspects of the innovation were flagged for adaptation during the final stages of preparing it for live implementation. These included terms of use, time zone configuration to reflect local time and date, safety and moderation protocols, the *need help now* feature, and the list of trigger words to flag posts indicative of potential risk.

**Conclusions:**

In the context of the COVID-19 pandemic and public health guidelines for social distancing, there is an increasing interest and need to leverage the internet and mobile technologies for delivering youth mental health services. As countries look to one another for guidance on how to navigate changing social dynamics, knowledge on how to utilize and adapt existing innovations across contexts is now more important than ever. Using a systematic approach, this study illustrates the methods, processes, results, and lessons learned on adapting a digital health innovation to enhance its local acceptability.

**International Registered Report Identifier (IRRID):**

RR2-10.2196/resprot.8810

## Introduction

### Digital Health Adaptations

Health service providers, community organizations, and consumers are increasingly looking toward leveraging digital health/eHealth innovations developed and tested in one part of the world (eg, country, state, region) for importation and use in their local communities. This approach can help reduce duplication of efforts and make better use of investments from the public, private, and philanthropic sectors of the community [1]. At the same time, little is known about the optimal processes involved in adapting digital health innovations when implementing them across geographical, cultural, and other types of contexts [1,2].

A *digital health (eHealth) adaptation* refers to a “systematic, purposeful, and collaborative process of making changes*”* to a digital health innovation to increase its “relevance and acceptability*”* for a local community [1]. Building on the adaptation models from the education, psychology, and technology fields, eHealth adaptation involves the consideration of factors such as language, culture, and context [1,3,4] when tailoring innovations for use in local settings. The ultimate purpose of adaptation is to increase the likelihood for an innovation to have value and impact for the population it is being adapted for. This is in alignment with the current guidelines for developing and evaluating complex interventions that highlight the importance of adapting interventions to local settings to improve their impact potential [5].

Indeed, within the broader mental health literature, previous studies have indicated that interventions originally designed for a population and adapted to the cultural and contextual needs of another population can yield better outcomes than providing those interventions to the same population without adaptation [2,6-8]. However, there are inconsistencies in terms of the extent of adaptations reported across studies [2]. There are also challenges in reviewing the effectiveness of adapted eHealth innovations given the limited consensus on guidelines, methods, and processes for adaptation and limited documentation on the adaptations undertaken by researchers when transporting or importing digital health innovations across contexts [1].

### The Horyzons Platform

In a previous paper, we reported on a digital health adaptation framework and research protocol for adapting Horyzons, an innovative digital mental health intervention that was originally developed and tested in Australia, in preparation for a pilot implementation study in Canada [1]. Horyzons is a web-based application powered by the Moderated Web-Based Social Therapy (MOST) system. MOST consists of interactive and strengths-focused psychosocial interventions, web-based social networking, and clinical and peer moderation. By tailoring the therapy content to target the treatment of specific conditions and adding any required code customizations, the flexible MOST platform enables the setting up of individual sites for a variety of mental health cohorts. Horyzons is a MOST-based intervention originally designed for cohorts of young people recovering from first-episode psychosis (FEP). The original version of Horyzons was developed iteratively over 30 months following participatory design principles and user-centered methodologies by an interdisciplinary team of experts in collaboration with young people receiving specialized early intervention (SEI) services for FEP [9].

Horyzons has been tested on a sample of 20 young Australian adults for its feasibility, acceptability, utility, and safety [9,10], and more recently, it has been pilot-tested in other international contexts [11]. There is also a randomized controlled trial of Horyzons [12] that was completed in 2018 and is currently undergoing data analysis. In addition, the MOST platform has been improved and adapted to address the needs of young people and their caregivers across the diagnostic spectrum [13-17]. Further details on the Horyzons platform and its core features are provided in our research protocol detailing phase 1 of our international research program [1].

### Objectives

The objectives of phase 1 were to assess the initial acceptability of Horyzons and adapt it in preparation for phase 2 pilot testing in Canada. In this paper, we report on the results of our phase 1 adaptation study.

## Methods

### Study Design and Setting

This study applied a mixed methods approach, combining descriptive quantitative and qualitative methods. The research took place in 2 SEI clinics for FEP, located in 1 urban and 1 small urban-rural setting in 2 Canadian provinces (Quebec and Ontario). Both programs provide a comprehensive range of services for young people diagnosed with FEP and follow best practice guidelines [18,19]. Ethics approval was first obtained from the ethics review board of the primary recruitment site before seeking and obtaining approval from the ethics review board of the secondary site.

### Participants

All participants (clinicians and patients) provided written informed consent before participating in the study. Eligibility criteria for patients were as follows: diagnosed with a psychotic disorder, within their first 3 years of treatment, currently in treatment, considered to be symptomatically stable and capable of participating in focus groups as judged by their primary treating clinician, 18 years of age or older, and the ability to speak and read in English. Eligibility criteria for clinicians were psychiatrists, case managers, or other health care professionals with a minimum of 2 years of experience working in the field of SEI for FEP and regularly involved in delivering services to youths with FEP.

### Adaptation Framework and Data Collection

The adaptation framework informed the data collection methods and processes. The framework is organized by 3 objectives and stages: (1) assess the initial perspectives of service users and providers of the eHealth intervention (before any modifications) following a brief orientation to the website, (2) assess the perspectives of the eHealth intervention following an extended exploration of the website, and (3) adapt the eHealth intervention on the basis of feedback from key stakeholder groups (while respecting its core therapeutic elements and principles and considering feasibility). Further details on the methods and processes of the eHealth adaptation framework are described in our previous protocol publication [1].

Data collection included a sociodemographic and technology use questionnaire, focus groups, and written feedback forms (Table 1). Sociodemographic and technology use questionnaires were used to ascertain the sociodemographic features of the participant sample (for both clinicians and patients) and to better understand their baseline access to and experience with technology.

**Table 1:** Summary of data collection activities.

Stage	Participants	Data collection activities
1	11 patients: 6 urban, 5 urban-rural	Sociodemographic and technology use questionnaire
	15 clinicians: 10 urban, 5 urban-rural	Exploration of beta version of the web portal and focus group discussion (90-120 min). Includes the following steps: Assigned to individual computer stations and provided user access Orientation to the web portal Individual exploration of the web portal Individual feedback forms, for example, general impressions; usefulness, safety, and support; design, layout, navigation; accessibility; and organizational capacities (the latter pertaining to clinicians only) Group discussion
2	6 patients: 4 urban, 2 urban-rural; 4 clinicians: 3 urban, 1 urban-rural	Personal extended exploration of beta version of the site, including the following steps: Up to 120 min over a 2- to 4-week duration from a personal computer, guided by a set of instructions on activities to complete; completion of the feedback form on personal impressions and suggestions for modifications and adaptations
3	4 clinicians: 2 urban, 2 urban-rural	With clinicians: individual consultation interview and follow-up meetings to review and complete recommended adaptations
	2 patients: 1 urban, 1 urban-rural	With patients: reviewing modifications and completion of the feedback form

In alignment with our eHealth adaptation framework, data collection was conducted in 3 iterative stages by a research assistant (LR). Clinicians and patients participated in all the 3 stages in separate groups. In the first stage, all participants were invited to complete the sociodemographic and technology use questionnaire, followed by an exploration of the web portal within the context of a focus group discussion (see Multimedia Appendix 1 for the visual setups of the group discussion). During the focus group, participants were asked questions on likes and dislikes of the platform, helpfulness of the content and features, how engaging the platform is to use, factors that could reduce motivation, platform safety, thoughts on the supports offered, design and layout, ease of use, difficult-to-understand words or expressions, and suggestions for adaptations (eg, language, metaphor, ease of use, content).

The second stage consisted of personal exploration of the platform. All participants were given log-in access to the platform. They were invited to explore its content and features over a 2- to 4-week duration on their personal computer, complete a set of activities on the platform, and fill out a personal feedback form regarding their comments and suggestions for adaptations. Feedback topics included items such as appropriateness of language; likes, dislikes, facilitators, and barriers; usefulness; safety; design and ease of use; therapeutic alignment; and organizational factors. Clinicians and patients were encouraged to explore 4 features, activities, or modules to evaluate (2 preselected by researchers, for clinicians these were preselected on the basis of their area of expertise, and 2 selected by the participants). Clinicians provided written feedback for each activity that included suggested adaptations, reasons why the adaptations were important, and general comments. Patients provided written feedback related to each activity regarding content; difficult-to-understand sentences or words; and likes, dislikes, and general comments. Patients were also asked additional questions regarding other activities they tried on the website, their comments on those other activities (eg, likes, dislikes, recommendations for change), and any additional comments or suggestions.

In the third stage, we conducted individual consultation interviews with clinicians to complete the recommended adaptations and interviews with patients to review the adaptations. This process was iterative in the sense that it involved back and forth communication with participants and revisions to arrive at a satisfactory version of the adaptations.

### Data Management and Analysis

Quantitative data from the sociodemographic and technology use questionnaires were analyzed using simple descriptive statistics (counts and percentages). In terms of data management, the qualitative data from the focus group discussions were recorded and transcribed verbatim and written feedback responses from the focus group and the extended exploration phase were organized into tables. The qualitative data were managed using Atlas.ti (version 7.5.6; ATLAS.ti Scientific Software Development GmbH), which supported the coding and analysis process. First, a preliminary coding framework was developed on the basis of the research objectives and initial review and discussion of the data by SL and LR. Once the coding framework was finalized (Multimedia Appendix 2), it was systematically applied to all the data by LR. A synthesis table of the data for each of the codes was then prepared per site (including the participant ID, data source, and verbatim citation) and themes were identified on the basis of patterns within the data (ie, topic mentioned by a minimum of 3 participants). The coding was also audited by a second research assistant who was not involved in the data collection and who was asked to assign codes to the extracted data. This audit process revealed minor discrepancies, which were discussed among the research teams to arrive at a consensus. A final review of the coding was then conducted by SL.

## Results

### Sociodemographic Characteristics

In total, 11 patients and 15 clinicians participated in this adaptation study. More than half of the patients were males (6/11, 55%) and between the ages of 19 and 25 years (6/11, 55%). The majority of patients reported completing a college-level education or higher (8/11, 73%); engaging in school, work, or caregiving activities (8/11, 73%); an annual income of less than Can $14,999 (approximately US $10,499) (9/11, 82%); and receiving specialized services for FEP for more than 2 years (7/11, 64%; see Table 2 for additional details on the sociodemographic characteristics of the patients).

**Table 2:** Sociodemographic breakdown of patients (total=11; urban=6; urban-rural=5).

Characteristics		Values, n (%)
**Gender**		
	Male	6 (55)
	Female	4 (36)
	Other	1 (9)
**Age (years)**		
	19-25	6 (55)
	26-37	5 (45)
**Race**		
	White	7 (64)
	Two or more races	3 (27)
	Hispanic	1 (9)
**Education (highest level completed)**		
	Less than high school	1 (9)
	High school	2 (18)
	College/vocational degree or diploma	5 (45)
	Bachelor’s degree	2 (18)
	Graduate diploma	1 (9)
**Length of receiving services at urban or urban-rural site**		
	0-6 months	4 (36)
	>2 years	7 (64)
**Current occupation**		
	Not in education, employment, or caregiving activities	3 (27)
	Working	5 (45)
	In school	1 (9)
	In school and working	2 (18)
**Annual household income, Can $ (US $)**		
	Under 14,999 (10,499) per year	9 (82)
	15,000-29,000 (10,500-20,300) per year	1 (9)
	50,000 (35,000) and above per year	1 (9)
**Current living situation**		
	Live alone	5 (45)
	Live with parent(s)/sibling(s)	5 (45)
	Live with partner and children	1 (9)
**Current relationship status**		
	Single	8 (73)
	In a relationship	2 (18)
	Legally married	1 (9)
**Access to a smartphone**		
	Yes	9 (82)
	No	1 (9)
	Other/sometimes	1 (9)

**Access to a home computer**		
	Yes	8 (73)
	No	3 (27)
**Use of the internet to search for mental health information, services, and support**		
	Once per week or less	8 (73)
	Once per week	1 (9)
	2 to 3 times per week	2 (18)
**Use of social media to communicate with others**		
	Yes	9 (82)
	No	2 (18)
**Frequency of use of social media**		
	Once per week or less	2 (18)
	2 to 3 times per week	2 (18)
	Daily	5 (45)
	N/A^a^	2 (18)
**Use of SMS text messaging to communicate with others**		
	Yes	8 (73)
	No	3 (27)
**Frequency of use of SMS text messaging**		
	2 to 3 times per week	1 (9)
	Daily	7 (64)
	N/A^a^	3 (27)
**Use of email to communicate with others**		
	Yes	9 (82)
	No	2 (18)
**Frequency of use of email**		
	Once per week or less	1 (9)
	Once per week	1 (9)
	2 to 3 times per week	2 (18)
	Daily	5 (45)
	N/A^a^	2 (18)
**To what extent do you feel competent in using a computer**		
	Not competent	0 (0)
	Somewhat not competent	0 (0)
	Neutral	4 (36)
	Somewhat competent	4 (36)
	Very competent	3 (27)
**To what extent do you feel competent in using social media to communicate with others**		
	Not competent	1 (9)
	Somewhat not competent	0 (0)
	Neutral	2 (18)
	Somewhat competent	5 (45)
	Very competent	3 (27)
**To what extent do you feel competent in searching the internet for mental health information, services, and supports**		
	Not competent	0 (0)
	Somewhat not competent	2 (18)
	Neutral	4 (36)
	Somewhat competent	4 (36)
	Very competent	1 (9)

^a^N/A: not applicable.

In terms of patient access, use, and perceived competence with technology (Table 2), the majority (9/11, 82%) had access to a smartphone and to a home computer (8/11, 73%). Only a few patients (3/11, 27%) reported using the internet to search for mental health information, services, and supports once per week or at least two to three times per week; more than half of the patients reported using SMS text messaging (7/11, 64%) daily and social media (7/11, 64%) daily or at least two to three times per week to communicate with others. Three patients reported not using SMS text messaging and 2 patients reported not using social media for communication purposes at all. In terms of perceived competence with technology, the majority of patients reported feeling at least somewhat competent with respect to using a computer (7/11, 64%) and using social media to communicate with others (8/11, 73%). Slightly less than half of the patients reported feeling at least somewhat competent searching the internet for mental health–related content (5/11, 45%), and the remaining patients reported feeling neutral (4/11, 36%) or somewhat not competent (2/11, 18%).

With regard to clinicians, the majority were women (12/15, 80%), between the ages of 26 and 56 years, had a master’s degree (9/15, 60%), and had a professional background in social work (7/15, 47%) or nursing (3/15, 20%). Their duration of time working in youth mental health services ranged from 2 to 16 years (see Table 3 for additional details on the sociodemographic characteristics of the clinicians).

In terms of clinician access, use, and perceived competence with technology (Table 3), the majority (14/15, 93%) had access to a smartphone and all had access to a home computer. A little over half of the clinicians (8/15, 53%) reported using the internet to search for work-related mental health information, services, and supports daily or at least two to three times per week. The majority reported using SMS text messaging (13/15, 87%) daily, and more than half reported using social media (10/15, 67%) daily to communicate with others. In terms of perceived competence with technology, approximately half of the clinicians (7/15, 47%) reported feeling somewhat competent when using a computer and social media, with 40% (6/15) feeling very competent using a computer and 27% (4/15) feeling very competent using social media to communicate with others. Approximately 60% (9/15) reported feeling very competent when searching the internet for mental health–related content, and the remaining 40% (6/15) indicated feeling somewhat competent with this activity.

**Table 3:** Sociodemographic breakdown of clinicians (total=15; urban=10; urban-rural=5).

Characteristics		Values, n (%)
**Sex**		
	Male	3 (20)
	Female	12 (80)
**Age (years)**		
	26-40	7 (47)
	42-56	8 (53)
**Race**		
	White	14 (93)
	Asian	1 (7)
**Education** **(highest level completed)**		
	College/vocational degree/diploma	3 (20)
	Bachelor’s degree	2 (13)
	Master’s degree	9 (60)
	Medical degree	1 (7)
**Professional discipline**		
	Psychology	1 (7)
	Psychiatry	1 (7)
	Human relations	1 (7)
	Occupational therapy	2 (13)
	Nursing	3 (20)
	Social work	7 (47)
**Length of time working in youth mental health services (years)**		
	2-5	4 (27)
	6-10	6 (40)
	11-16	5 (33)
**Access to a smartphone**		
	Yes	14 (93)
	Other/sometimes	1 (7)
**Access to a home computer**		
	Yes	15 (100)
**Use of the internet to search for work-related mental health information, services, and supports**		
	Once per week or less	7 (47)
	2 to 3 times per week	2 (13)
	Daily	6 (40)
**Use of social media to communicate with others**		
	Yes	12 (80)
	No	3 (20)
**Frequency of use of social media**		
	2 to 3 times per week	2 (13)
	Daily	10 (67)
	N/A^a^	3 (20)

**Use of SMS text messaging to communicate with others**		
	Yes	15 (100)
**Frequency of use of SMS text messaging**		
	Once per week or less	1 (7)
	2 to 3 times per week	1 (7)
	Daily	13 (87)
**Use of email to communicate with others**		
	Yes	14 (93)
	No	1 (7)
**Frequency of use of email**		
	Once per week or less	4 (27)
	2 to 3 times per week	3 (20)
	Daily	7 (47)
	N/A^a^	1 (7)
**To what extent do you feel competent in using a computer**		
	Not competent	0 (0)
	Somewhat not competent	1 (7)
	Neutral	1 (7)
	Somewhat competent	7 (47)
	Very competent	6 (40)
**To what extent do you feel competent in using social media to communicate with others**		
	Not competent	1 (7)
	Somewhat not competent	2 (13)
	Neutral	1 (7)
	Somewhat competent	7 (47)
	Very competent	4 (27)
**To what extent do you feel competent in searching the internet for mental health information, services, and supports**		
	Not competent	0 (0)
	Somewhat not competent	0 (0)
	Neutral	0 (0)
	Somewhat competent	6 (40)
	Very competent	9 (60)

^a^N/A: not applicable.

### Perspectives on the Digital Health Innovation

The results from the focus groups and written feedback from the extended explorations are organized according to the following themes: (1) appreciating the therapeutic approach and relatability of Horyzons; (2) diverging opinions on design, layout, and ease of navigation; (3) being concerned about implementation; and (4) providing suggestions for changing content and features. The following sections provide further details on each of these themes with illustrations through participant quotes. Quotes are labeled with ID codes that represent the stakeholder group (ie, patient, P, or clinician, C). Given the small sample size, we removed gender and site-level identification to protect the anonymity of the participants.

#### Appreciating the Therapeutic Approach and Relatability of Horyzons

Overall, the majority of patients (10/11, 90%) and all clinicians (15/15, 100%) expressed appreciation of the platform in terms of its therapeutic approach (eg, related to content, activities, social media features) and relatability for young people, including those that have experienced FEP. For example, in terms of relatability, patient participants stated:

The website does a nice job of outlining scenarios that are very relatable as well as steps for how to deal with these different instances.

P1

I enjoyed reading people’s stories because it is from a trusted site and it was interesting to see stuff I could relate to from their story.

P2

Similarly, clinicians mentioned that the stories of characters presented on the website “provided a relatable way to deliver psychoeducation about psychosis,” (C1) and that the content is relatable for youth:

It’s more of a general site for everybody, which I think is good. And I get the impression that these are general topics that young adults and young adolescents anyway would talk about.

C2

Positive comments about Horyzons were made by both clinicians and patients in reference to its content, strengths-focused activities, and social media features. In terms of content, clinicians commented that the platform was informative, for example:

As a platform for information, I think it’s fantastic and I think our client, from entry to exit, could easily benefit from this.

C3

Patient participants highlighted their interest in topics addressed in the website:

I liked the content itself. I find it interesting topics and stuff.

P3

Across both stakeholder groups, participants expressed appreciation for how content was presented and organized, in relation to tips, things to do on the website, and multimedia:

I appreciate the videos and audio tracks, not everyone wants to read a lot of material. It is easier to follow audio tracks also for certain types of exercises.

C4

I found the tips helpful.

P4

I found that since there are lots of [activities], like it can help many people.

P5

Clinician and patient participants also linked the therapeutic value of the platform to evidence-based psychosocial therapies such as cognitive behavioral therapy and mindfulness therapy:

It’s like CBT! … tips to change your behav[iour], to improve your wellbeing.

P7

Good ‘training’ on affective regulation, mindfulness in an understandable and user-friendly way.

C1

In terms of the strengths-based approach, patients and clinicians highlighted the personal strengths card sorting activity conducted at the beginning of the website intervention:

I can see how this would make someone feel pretty positive about themselves … because there’s so many to choose from, like ‘Wow! I have way more than 5 top strengths!

C5

Several of the participants (patients and clinicians) reacted favorably toward the social media and community features of the platform and expressed appreciation of how they resembled mainstream social media, such as Facebook (P8; C6) and how the website includes clinical moderation with peer networking:

Mixing the community part with the like helping part. I think that’s a good idea.

P5

Patient participants highlighted the value of these features in relation to accessing peer support:

Lets people know that other people experience similar things.

P10

Being able to talk to other people that have or are going through similar things.

P11

We can exchange with others on certain subjects/share stories.

P7

In addition, clinicians commented that Horyzons could be a useful resource when patients start receiving specialized services for FEP in addition to transitions between services. For example, one clinician explained how the site could help patients stay engaged in their treatment:

The stories and explanations are great and could…encourage newly admitted FEP clients to maintain their treatment, alliance building, etc.

C3

#### Diverging Opinions on Design, Layout, and Ease of Navigation

Participants across both groups reported contradicting opinions on the design, layout, and navigation. For instance, some appreciated the calm and neutral look of the site. One patient explained:

I like that there’s nothing that’s too distracting on it

P3

Others would have preferred a more colorful design or sounds to certain features for a more dynamic and interactive experience. Only 1 patient raised general issues with respect to navigating the site, in contrast to several clinicians who expressed challenges navigating information on the site. For example, clinicians expressed that there was a lot of text to navigate on the platform, that the exploratory navigation style of the platform may lead to some users disengaging from the site, and that the organization of content could be better organized:

It would be more useful for the content to be organized under clear headings rather than have to sift through what has been posted on the main wall.

C7

#### Being Concerned About Implementation

Clinicians raised several concerns about its implementation. These concerns pertained to patient safety, clinical moderation, and internet connectivity. For example, clinicians raised concerns about the respectful use of the site:

I wonder what the “café” comments and other comments from users will look like. Will they be respectful? There could be some occasional problems.

C1

Clinicians also voiced concerns about users witnessing others on the site going through a crisis, crisis response management, integration of moderation into clinical workflow, and new roles and expectations (eg, in relation to web-based moderation and web-based crisis management response). Regarding the latter, some clinicians were concerned that if web-based moderation of the site became a part of their workday, it would take away from their duties as a case manager:

I think there is a certain amount of time that a clinician will have to look through it. So, whatever time they are spending doing that, it is time they are not actually doing case managing…personally, for me, it is not the type of work I would do with my clients.

C7

Clinicians also expressed concern regarding technology infrastructure. Participants from the urban-rural setting experienced navigation and uploading difficulties due to a very slow internet connection. They highlighted that having internet connectivity issues was common in their setting and something that will need to be considered in a live implementation scenario:

Our internet is not going to be like it is in the city… but that’s something that they’re gonna have to think about, right? Because it is really really different access here.

C9

Patients also raised concerns about access and safety. They reported that some individuals may not want to connect with others on the web, “they might prefer to deal with their problems more privately” (P1); might have limited access to the technology needed to participate on the site, “access to the internet/computer capable of using the site” (P11); or might be deterred from using the site due to their psychosis symptoms, “fear of being tracked about what is being said or done (paranoia for some youth)” (P11) or due to concerns “about online bullying” (P11). They offered several strategies to promote engagement with the platform, including having its use:

Encouraged by their doctor or peers.

P1

Making the site cell phone accessible.

P11

Putting stories on there about successful recovery (normal people and celebrities alike).

P11

#### Recommending Changes to Content and Features

In terms of recommendations for adaptations, patient (4/11, 36%) and clinician (13/15, 86%) participants provided various suggestions. Mostly, these pertained to making Canadian-specific adaptations to content on finding work, study, and volunteer opportunities (job interviews, job-finding tools, job hunting and study searching, work, study, and life) and content on postdischarge care (finding a doctor, paying for postdischarge support, and support after discharge). They also recommended having the audio tracks read by individuals with a Canadian accent (vs Australian) and adding content pertaining to physical health (eg, adding a section on sleep hygiene), medication use and side effects, aptitude tests and self-rating scales, information on nondistressing psychosis, and links to other websites and resources:

Definitely agree that there needs to be more resources connected to the website. So, you know, it’s not just one mental health issue, it’s many issues, so it’s best to know what’s out there.

P12

In terms of the features of the site, clinicians recommended reducing the volume of text and adding more audio tracks to deliver the content, given that youths recovering from FEP can have:

residual cognitive difficulties which could limit their ability to read through text for extended time periods.

C8

#### Adapting the Digital Health Innovation

Subsequent to the data collection phases of this study, we proceeded to develop the content for the module adaptations, especially in relation to employment, education, and postdischarge. This process was conducted iteratively (ie, back and forth communications and reviews until a satisfactory outcome was achieved) in consultation with four of the clinician participants (2 urban and 2 urban-rural) and two of the patient participants (1 urban and 1 urban-rural). With respect to the topics of employment, study, and volunteer opportunities, modifications were made to the following sections on Horyzons: (1) job-finding tools, (2) job hunting and study searching, (3) job interviews, and (4) work, study, and life. For example, modifications were made to ensure that local organizations and government agency services, resources, and links replaced the Australian ones to reflect the local provincial and municipal job market. Moreover, some of the content was adapted to Canadian norms, for example, the *do’s and don’ts* of what is included in a resume.

With respect to the topic of postdischarge, modifications were made to the entire content, and the sections were named as follows: (1) getting provincial health insurance, (2) finding a family doctor, and (3) postdischarge follow-up. Clinician participants suggested that the text in these modules be presented using flowcharts and tables, and they participated in the development and visual representation of this content. For example, for the module on postdischarge, one clinician suggested a table to organize information that provides patients with a brief description of the 3 types of postdischarge follow-up care that are available to them depending on their needs.

Once a preliminary version of the adaptations was completed, it was shared with the patient participants along with a feedback form and a brief survey. One patient participant from each site completed the feedback form and the survey, and both expressed that they perceived the job-related modules to be helpful and informative. The patient participants also provided additional recommendations at this stage, such as modifications to the sample resume in terms of using “more specific or detailed information” (P1); adding an example of *cold emailing* to potential employers; expanding the section on what the provincial employment sector “offers to help seek employment” (P11); adding “more information on what is deducted from a pay,” “what might constitute diminished capabilities at a place of work,” and “how to balance work, school, and life so as to not get over stressed and have a relapse” (P11).

With regard to the postdischarge modules, the patient participants had limited feedback. One of the participants suggested including more information about transitioning from disability support to gainful employment and how this relates to health insurance. This same participant also commented on needing more information pertaining to which type of postdischarge setting (eg, family doctor vs secondary outpatient psychiatry) would be most suitable for different circumstances.

Once the content of the adaptations was finalized, a separate copy of the Horyzons platform was created for Canadian use in which the adaptations were made, herein referred to as HoryzonsCa. The platform has an inbuilt authoring system, so that those responsible for localizing content can simply log on to the system and make the changes themselves, with limited need for technical support. We then began preparing for the live pilot implementation study of HoryzonsCa. We decided to focus the pilot study on the urban site, given the issues with internet connectivity at the urban-rural site. This issue may be something for the research team to consider for future projects in community settings that have limited infrastructure in terms of internet connectivity. The preparations for the pilot study resulted in additional modifications to the following: terms of use, setting the time to match our time zone (ie, Eastern Standard Time), making the *need help now* button more apparent by changing the font color to red and adapting the content of the *need help now* page to the local context.

MOST contains a mechanism whereby client newsfeed postings containing problematic words, as contained in a system word list, are automatically blocked from being posted. This list of words consists of both swear words and danger words (ie, words indicating that the user is at risk). Postings with swear words are blocked, and the user is prompted to provide an appropriately reworded version of the post. Posts containing danger words are automatically sent to a moderation quarantine, where they are assessed by a moderator and either deemed okay and released to the newsfeed or deemed problematic, prompting a check in with the user. We translated the Australian list of words to French and made any other culturally required modifications even though the implementation was planned to be in English. This is because many of the youths seen in the program are bilingual, with their mother tongue being French. As such, it was considered that some of these individuals might naturally revert back to their mother tongue during crisis situations and moments of disinhibition. Thus, to increase the safety of the platform, we added translated French words to the trigger list. We also made changes to the moderation interface of the platform, including the contact list, safety check and supervision roster, and the structure and process for the moderators to document their notes to fit with local practices (see Table 4 for a summary of the adaptations made to the Horyzons platform).

**Table 4:** Summary of adaptations.

Original features	Participant feedback	Adapted version
Job-finding tools module	Keep the structure and integrate the local content	Canadian *do’s and don’ts* of writing a resume and a cover letter A sample resume and cover letter
Job-hunting and study-searching module	Keep the structure and integrate the local content	Description and links to provincial and local job-seeking sites, government agencies, employment centers, major employers (eg, retail and fast food), LinkedIn, recruitment agencies, student internships (for urban areas only), web-based resources and career guides, career guidance professionals, and finding volunteer work Deleted one section on a training and university program only relevant to Australia
Job interviews module	Keep the structure and integrate the local content	No modifications
Work, study, life module	Keep the structure and integrate the local content	Description and link to the provincial employment guide Provincial and local answers to the drop-down menu of questions related to pay and conditions, health, and work
Finding a doctor module	Completely adapt the content and visual display of information to make it locally relevant and easy to navigate	New module name: Finding a family doctor Flowchart with *yes* and *no* questions with provincial content and links
Paying for postdischarge support module	Completely adapt the content and visual display of information to make it locally relevant and easy to navigate	New module name: Getting provincial health insurance Flowchart with *yes* and *no* questions with provincial content and links
Support after discharge module	Completely adapt the content and visual display of information to make it locally relevant and easy to navigate	New module name: Postdischarge follow-up Local postdischarge follow-up options detailed in a visual format
General use and safety features	—^a^	Adapted terms of use Changed to local time zone Enhanced the *need help now* button and integrated local content
Moderation features	—	Translated Australian list of problematic words to French because local youths taking part in the pilot phase may be bilingual, with their mother tongue being French Adapted the following to local content and practices: contact list, safety check, supervision roster, and structure and process for moderators’ notes

^a^Participants did not give specific adaptation feedback on the topic.

## Discussion

### Principal Findings

Overall, the HoryzonsCa beta version was well received by both patient and clinician participants. In particular, they appreciated the strengths-based therapeutic approach of the platform and considered the content and social media features to be supportive of the recovery process. Their interest and positive comments about this digital health innovation provide us with the validation for moving forward with a live pilot implementation study of HoryzonsCa. Across the various stages of the adaptation study, both clinician and patient participants provided different types of comments, suggestions, and questions. Feedback offered insights into attitudes toward the innovation, potential barriers to its implementation, and adaptations to make before piloting the intervention within a Canadian context. Some of the suggestions from participants in this study are consistent with those reported in previous research on Horyzons (eg, making the intervention accessible via mobile devices) [11]; concurrently, many of the suggestions for adaptations and improvement are novel. This may be due to the qualitative nature of our process, the elicitation of feedback from multiple stakeholder groups (ie, clinicians in addition to patients), the multiple opportunities given to reflect and contribute, the active engagement of participants in the adaptation process, and the factors related to context (eg, different characteristics of the employment and health sector). In addition, many of our patient participants had sociodemographic characteristics that may have facilitated receptivity to Horyzons, for example, several had high levels of education, which has been associated with active use of the internet [20], seeking medical information on the web [21], and the depth and breadth of engagement with web-based interventions [22] among those with psychosis and other psychotic disorders.

Beyond the content-specific modifications for Canadian applicability, this adaptation study also highlighted several issues for future consideration. First, we learned of the utility of having a framework to guide the adaptation process. The digital health innovation adaptation framework [1] that we used was feasible to apply and provided a clear pathway for the adaptation process. We also learned that digital health adaptation processes should incorporate multiple opportunities over time for users to provide feedback on how a digital health innovation can be optimized to address their needs. However, it is also important to consider that tailored adaptations of a digital health innovations across multiple settings and population groups may raise challenges for managing the technology infrastructure on the back end; this could be an area warranting consideration in future digital mental health innovation research.

We also learned from this study that a multiple stakeholder consultation approach to digital health adaptation can create challenges in terms of meeting a diverse set of expectations. We balanced this challenge by prioritizing adaptations that were raised by multiple participants (ie, patterns) and through collective reflection on which adaptations were necessary before live implementation and which suggestions for adaptations could be considered as secondary. It is also important to note that none of the suggestions that were made jeopardized the core features of the intervention (eg, peer support or clinician moderation) nor its theoretical underpinnings (eg, self-determination theory).

Second, it is important to consider the universal accessibility of digital health innovations, including, for example, accessibility to individuals with visual, cognitive, and physical impairments. The latest version of the platform has begun to address some of these issues, for example, by reducing the amount of text and conveying information through visual media, such as comic illustrations. Third, it is important to consider that adaptations to a digital health innovation must align with jurisdictional guidelines for privacy and security. Fourth, as several participants outlined the lack of access to technology infrastructure as a barrier, it is important to optimize or configure the intervention for ease of navigation and access by communities living in urban-rural, rural, and remote settings with limited internet speed.

Finally, it is important to consider workforce capacity, training, and readiness in terms of providing blended approaches to care (eg, case management in person and on the web). With respect to the latter, the concerns raised by clinicians regarding moderating the site may be linked to limited knowledge and experience with using technology-based interventions and services. This lack of experience highlights the ongoing need for continuing education and training on digital health for the mental health workforce [23]. Aligned with this, it is also important to consider whether clinical training materials to implement digital health innovation need to be adapted for use in a specific context. Furthermore, there may be a need to consider new models for moderating digital health innovations that combine the human resource capacity across a range of stakeholders (eg, case managers, peer support workers, clinician moderators).

### Conclusions

With the majority of young people now using the internet, social media, and mobile devices, the use of technology is an important avenue for supporting recovery in young people experiencing FEP. Research on the use of technology to deliver psychosocial interventions is emerging within the field of early intervention for psychosis [24-27]; however, less attention has been given to how digital health innovations can be adapted across settings. Our study addresses this gap and contributes to the growing field of implementing digital health innovations within the context of mental health service delivery. Specifically, this adaptation study provides insights into the processes and outcomes of adapting and testing an existing digital health innovation for use in different contexts and health care settings. Using a systematic approach, this study identified specific aspects of a digital health intervention perceived by clinicians and patients as being important to adapt to enhance the contextual implementation and acceptability of the intervention. Our next plan is to test the acceptability, safety, and potential benefits (eg, clinical efficacy) of the adapted platform using a live version of the site with a sample of 20 to 25 participants over an 8-week follow-up period, with access to clinician and peer support moderators. Finally, in the context of the COVID-19 pandemic and public health guidelines for social distancing, there is an increasing interest and need to leverage internet and mobile technologies for delivering youth mental health services. As countries look to one another for guidance on how to navigate the changing social dynamics, knowledge on how to utilize and adapt existing innovations across cultural contexts is now more important than ever. Our study provides insights into the processes and methods that can be used to prepare for transporting a digital health innovation across settings and geographical contexts and lessons learned through this process.

## Appendix

Multimedia Appendix 1 Focus group room setup at the urban-rural site. Focus group room setup at the urban site.

Multimedia Appendix 2 Coding framework.

## Abbreviations

FEP: first-episode psychosis
MOST: Moderated Web-based Social Therapy
SEI: specialized early intervention
