# Supplementary material for: Adaptation of a Digital Health Innovation to Prevent Relapse and Support Recovery in Youth Receiving Services for First-Episode Psychosis: Results From the Horyzons-Canada Phase 1 Study
Source: JMIR Form Res. 2020 Oct 29;4(10):e19887. doi: 10.2196/19887 (PMC7661238; doi:10.2196/19887)
Supplement: Multimedia Appendix 1 [file formative_v4i10e19887_app1.pdf]

Multimedia Appendix 1

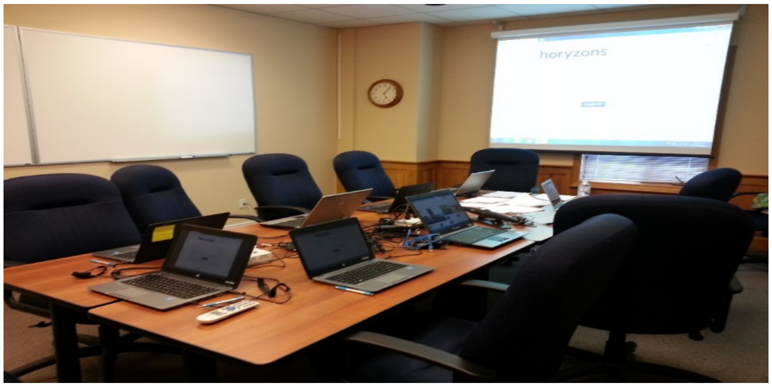

Figure 1. Focus Group Room Set-Up at the Urban-Rural Site

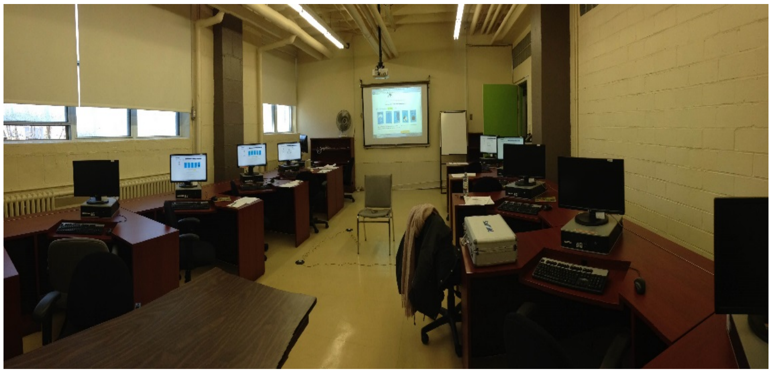

Figure 2. Focus Group Room Set-Up at the Urban Site
