# Supplementary material for: Adaptation of a Digital Health Innovation to Prevent Relapse and Support Recovery in Youth Receiving Services for First-Episode Psychosis: Results From the Horyzons-Canada Phase 1 Study
Source: JMIR Form Res. 2020 Oct 29;4(10):e19887. doi: 10.2196/19887 (PMC7661238; doi:10.2196/19887)
Supplement: Multimedia Appendix 2 [file formative_v4i10e19887_app2.pdf]

Multimedia Appendix 2

Coding Framework

Sub-codes
1. Likes<br>2. Dislikes<br>3. General comments/questions
1. Elements of the platform considered to be useful/helpful<br>2. Elements of the platform considered to be not useful/ helpful<br>3. Elements of the platform considered to be missing that would make the platform 'more' useful/helpful
1. Existing elements of the platform that enhance safety/ support<br>2. Elements of the platform that are considered to be missing that would make the platform safer and more supportive<br>3. Safety /support questions<br>4. Safety/support concerns<br>5. Issues/concerns/questions about moderation
1. Likes<br>2. Dislikes<br>3. Navigation difficulties<br>4. Suggestions for changes to layout/navigation
1. Elements of the platform considered to be accessible<br>2. Elements of the platform considered to be difficult to use/ inaccessible/questions
1. Content to be added<br>2. Content to be removed or changed<br>3. Language/terms/slang/expressions<br>4. Australian specific cultural/contextual elements that do not apply to Canadian culture/context
1. Capacity issues/concerns in implementing/moderating the platform<br>2. Therapeutic practices
1. Module – Clinician<br>2. Module – Patient<br>3. Site tracking – Clinician<br>4. Site tracking - Patient
